# DNA-enabled fluorescent-based nanosensors monitoring tumor-related RNA toward advanced cancer diagnosis: A review

**DOI:** 10.3389/fbioe.2022.1059845

**Published:** 2022-12-01

**Authors:** Fengqi Dong, Weizhen Yan, Wuqi Dong, Xiaofei Shang, Yanli Xu, Wei Liu, Yunkai Wu, Wenmei Wei, Tingting Zhao

**Affiliations:** ^1^ School of Basic Medical Sciences, Biopharmaceutical Research Institute, Research and Engineering Center of Biomedical Materials, Anhui Medical University, Hefei, China; ^2^ The First School of Clinical Medicine, Anhui Medical University, Hefei, China

**Keywords:** nanosensors, tumor-related biomarkers, RNA detection, cancer diagnosis, DNA-enabled fluorescent probe

## Abstract

As a burgeoning non-invasive indicator for reproducible cancer diagnosis, tumor-related biomarkers have a wide range of applications in early cancer screening, efficacy monitoring, and prognosis predicting. Accurate and efficient biomarker determination, therefore, is of great importance to prevent cancer progression at an early stage, thus reducing the disease burden on the entire population, and facilitating advanced therapies for cancer. During the last few years, various DNA structure-based fluorescent probes have established a versatile platform for biological measurements, due to their inherent biocompatibility, excellent capacity to recognize nucleic and non-nucleic acid targets, obvious accessibility to synthesis as well as chemical modification, and the ease of interfacing with signal amplification protocols. After decades of research, DNA fluorescent probe technology for detecting tumor-related mRNAs has gradually grown to maturity, especially the advent of fluorescent nanoprobes has taken the process to a new level. Here, a systematic introduction to recent trends and advances focusing on various nanomaterials-related DNA fluorescent probes and the physicochemical properties of various involved nanomaterials (such as AuNP, GO, MnO_2_, SiO_2_, AuNR, etc.) are also presented in detail. Further, the strengths and weaknesses of existing probes were described and their progress in the detection of tumor-related mRNAs was illustrated. Also, the salient challenges were discussed later, with a few potential solutions.

## Introduction

**FIGURE 1:**
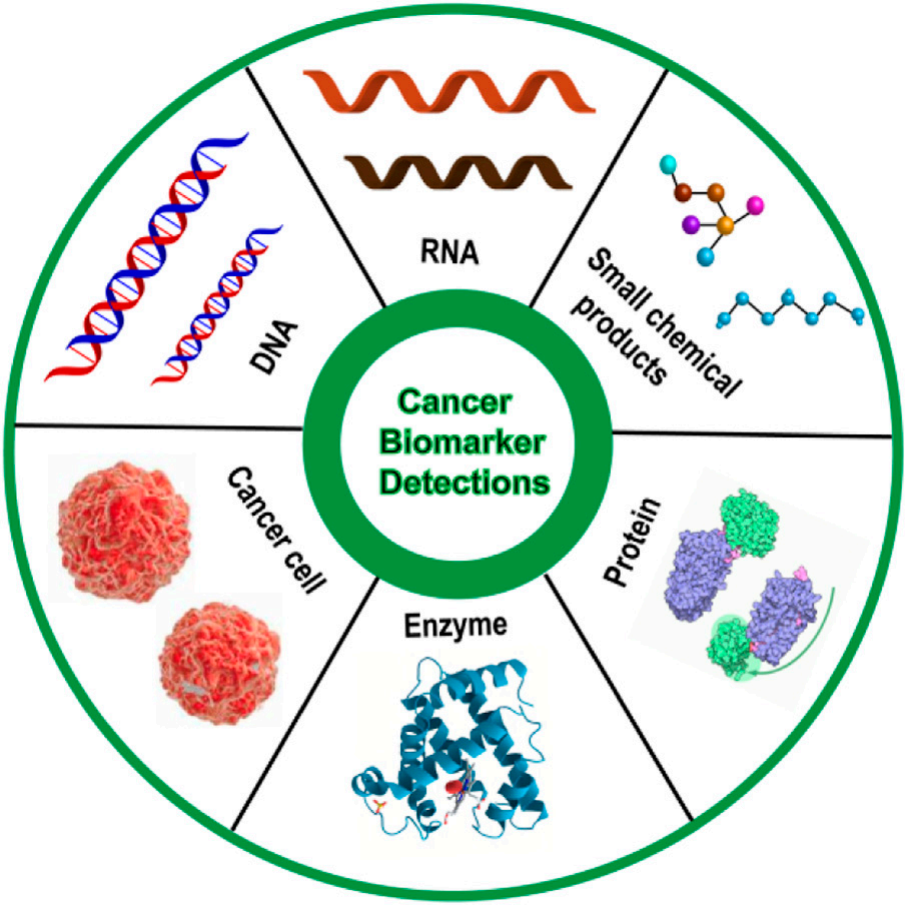
Schematic diagram of various cancer biomarkers.

Cancer is a complex disease characterized by neoplastic proliferation, atypia, and metastasis, holding high mortality all over the world (Duffy, 1996; Alvarez-Breckenridge et al., 2009; Gao et al., 2017). It is hard to diagnose cancer at an early stage, and there are limited treatment options and no effective drugs for advanced cancer, thus cancer mortality rate remains high. As an important tumor indicator for the early diagnosis of cancer, accurate detection of tumor biomarkers is capable of increasing the diagnosis rate, and then reducing cancer mortalit (Brown et al., 2021). Tumor biomarkers are usually present in the tumor tissue or serum of cancer patients, containing a variety of molecules, such as DNA, mRNA, transcription factors, enzymes, metabolites, and cell surface receptors (Wu and Qu, 2015) (Figure 1). As a tumor biomarker, RNA possesses diagnostic value, prognostic value, and predictive value in the field of oncology, which can not only provide early diagnosis of tumors, treatment risk assessment, and prediction of treatment response, but also can be used as a potential target for drug design, therefore, it is of great significance for the detection of RNA (Mayeux, 2004; Mehta et al., 2010). This review mainly introduces the detection of various mRNA by DNA-based fluorescent probes, such as TKI mRNA, hTR, surviving mRNA, MicroRNA, p21 mRNA, etc, (Table 1).

**TABLE 1:** The relationship between RNA type and diseases.

RNA type	Disease	Sequence (5′-3′)	Reference
TK1 mRNA	Most cancers	TGA​TCA​AGT​ATG​CCA​AAG​ACA​CTC​GCT​ACA	Yang et al. (2015)
p21 mRNA	Most cancers	UCG​GCU​CCC​CAU​GUG​UCC​U	Fan et al. (2019)
p53 mRNA	Most cancers	GCU​UUG​AGG​UGC​GUG​UUU​GUG​C	Fan et al. (2019)
c-myc mRNA	Most cancers	CCU​CAA​CGU​UAG​CUU​CAC​CAA	Jiang et al. (2018)
VEGF mRNA	Tumor and vascular proliferative diseases	CAU​CAC​CAU​GCA​GAU​UAU​GCG	Dang et al. (2019)
MicroRNAs	numerous solid tumors cardiovascular and autoimmune diseases	TAG​CTT​ATC​AGA​CTG​ATG​TTG​A	Ou et al. (2018)
hTR	Most cancers	TTT​TGT​CTA​ACC​CTA​ACT​GAG​AAG	Ning et al. (2017)
surviving mRNA	breast cancer	CAGCCCTTTCTCAA	Qiao et al. (2011)
Cyclin D1 mRNA	breast cancer	ATCTACACCGACAACTC	Qiao et al. (2011)

Cells contain a variety of biomolecules and a number of complex structures that make up the complexity of the cell (Hill and Meininger, 2012). As the complex intracellular environment may lead to feeble output and erroneous signals, the detection of these biological small molecules remains challenging (Fan et al., 2019). Molecular biology, microarray analysis (Clark et al., 2002), real-time polymerase chain reaction (RT-PCR) (Karbownik et al., 2005) and Northern hybridization (Pall et al., 2007) are the most commonly used techniques *in vitro* assays for mRNA detection, which have wide detection limits and high accuracy, yet existing shortcomings such as complex operation and time-consuming. For example, RT-PCR requires professional training to design primers in order to avoid the production of false positive signals. The ability of DNA-based fluorescent probes to identify nucleic acid, non-nucleic acid targets, easy synthesis, and chemical modification has attracted growing attention (Ebrahimi et al., 2020). Molecular beacon is the most common DNA fluorescent probe, which is simple, fast, and sensitive to achieve the detection of biological small molecules in the cell lysate, whereas it is difficult to enter the cell to achieve *in situ* detection of intracellular biological small molecules. In addition, due to the complex environment in the cell, even if it enters the cell with the help of transfection reagent, DNase I can cut off single-stranded and double-stranded DNA, resulting in the generation of false positive signals (Chen et al., 2007). Therefore, it is crucial to develop a probe that can stably *in situ* detect target inside cells.

For the purposed of avoiding false positive signals caused by the complex intracellular environment, nanomaterials emerge in the vision. As a result of the protective effect of nanomaterials on nucleic acid sequences, they are capable of maintaining stability in the complex intracellular environment (Seferos et al., 2009). In addition, when the DNA probe is connected to the surface of the nanomaterial, it can enter the cell with the help of endocytosis. Compared with the nucleic acid sequence probe alone, it has been greatly improved, not only protecting the probe but also allowing stable entry into the cell without the help of transfection reagents to achieve *in situ* detection in the cell. Furthermore, with the rapid emergence of nanotechnology, nanostructures have been widely used for reliable and robust signal amplification, improving the sensitivity of biosensors and producing higher precision. Recently, various nanomaterials including AuNP (Sun et al., 2019), GO (Shi et al., 2016), AuNR (Ma et al., 2017), MnO_2_ (Ou et al., 2018), ZnO (He et al., 2017), SiO_2_ (Zheng et al., 2017), and UCNP (Ding et al., 2016) have been used for *in situ* detection of intracellular mRNA (Table 2). The diversity of nanoprobes is constituted of nanomaterials possessing unique physical and chemical properties, whose application has highly innovated the traditional nucleic acid probe and pushed the study of DNA fluorescent probes to a climax. Apart from nucleic acid nanoprobes for *in situ* detection of intracellular tumor-related RNA, the electrochemical probes with the help of nanomaterials have also been greatly developed, which greatly improves the sensitivity of probes, making ultra-trace detection of tumor-related RNA a reality (Zhang et al., 2015; Gong et al., 2019). However, the electrochemical probes are usually used for detection of biological small molecules *in vitro*, and there are few reports of *in situ* detection of biological small molecules in cells. It remains a problem that researchers need to overcome. Therefore, this highly sensitive electrochemical probe to the *in situ* detection of cells still need to be explored, which will be a huge improvement for the detection of traces amount of tumor biomolecules.

**TABLE 2:** Different types of DNA-based fluorescent probes.

Materials	Detection limit	Mechanism	Stability in cell	Cell *in situ* detection	Sample	Excitation/Emission
Molecular Beacon (Ratajczak et al. (2018)	26 nM	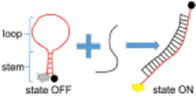	Poor	yes	survivin mRNA	520 nm/548 nm
Electrochemiluminescence (Zhang et al. (2015))	0.83 fM	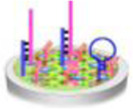	/	/	miRNA-21	/
AuNPs modified nucleic acid sequence (Yang et al. (2015))	not reported	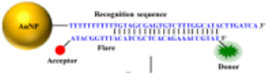	Excellent	yes	TK1 mRNA	488 nm/520 and 580 nm
GO modified nucleic acid sequence (Jiang et al. (2018))	0.26 nM	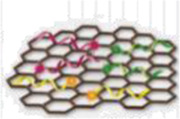	Excellent	yes	C-myc mRNA	550, 488, and 648 nm/570, 520, and 670 nm
	1.04 nM				TK1 mRNA	
	1.15 nM				Actin mRNA	
rGO modified nucleic acid sequence (Fan et al. (2019))	0.46 nM	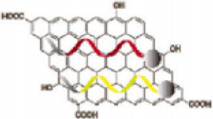	Excellent	yes	p21 mRNA	488 and 633 nm/520 and 660 nm
	0.71 nM				p53 mRNA	
MnO_2_ modified nucleic acid sequence (Ou et al. (2017))	1 nM	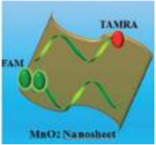	Excellent	yes	TK1 mRNA	488 nm/520 and 580 nm
AuNR modified nucleic acid sequence (Ma et al. (2017))	0.011 amol/ngRNA	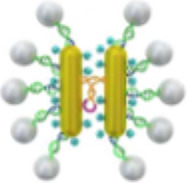	Excellent	yes	miRNA-21	/
SiO_2_ modified nucleic acid sequence (Yuan et al. (2017))	1.3 nM	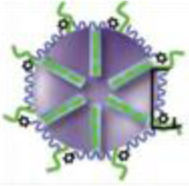	Excellent	yes	survivin mRNA	545 nm/580 nm
ZnO modified nucleic acid sequence (He et al. (2017))	1 fM	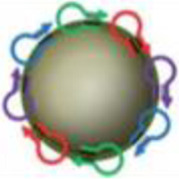	Excellent	yes	survivin mRNA	488 nm/526 nm
UCNP modified nucleic acid sequence (Ding et al. (2016))	1.1 nM	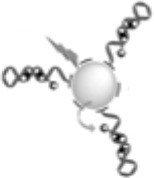	Excellent	yes	TK1 mRNA	540 nm/655 nm
QD modified nucleic acid sequence (Lin et al. (2021))	5.5 nM	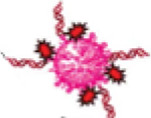	Excellent	yes	survivin mRNA	405 nm/670 and 720 nm

Due to unique physical and chemical properties, some nanomaterials can realize the integration of detection and treatment. For example, AuNP possesses excellent thermal conductivity that laser irradiation leads to the rise of temperature and then heats tumor cells to death, which is utilized for photothermal therapy (Zhang et al., 2019). SiO_2_ is used in chemotherapy because of its unique pores with good drug loading efficiency and drug protection ability (Heleg-Shabtai et al., 2016). It has been reported that using the drug loading characteristics of G and C bases to carry drugs, drug molecules can be inserted into the G and C base pairs and quench the fluorescence of the drug (Ma et al., 2016). When the nanoprobe enters the cell, the hydrogen bond between G and C is opened to realize the release of drugs and the integration of *in situ* detection and chemotherapy. This review provides a systematic introduction to the development of DNA fluorescent probes and the principles of nanoprobe design.

## DNA-based fluorescent probes

### Molecular beacons

**FIGURE 2:**
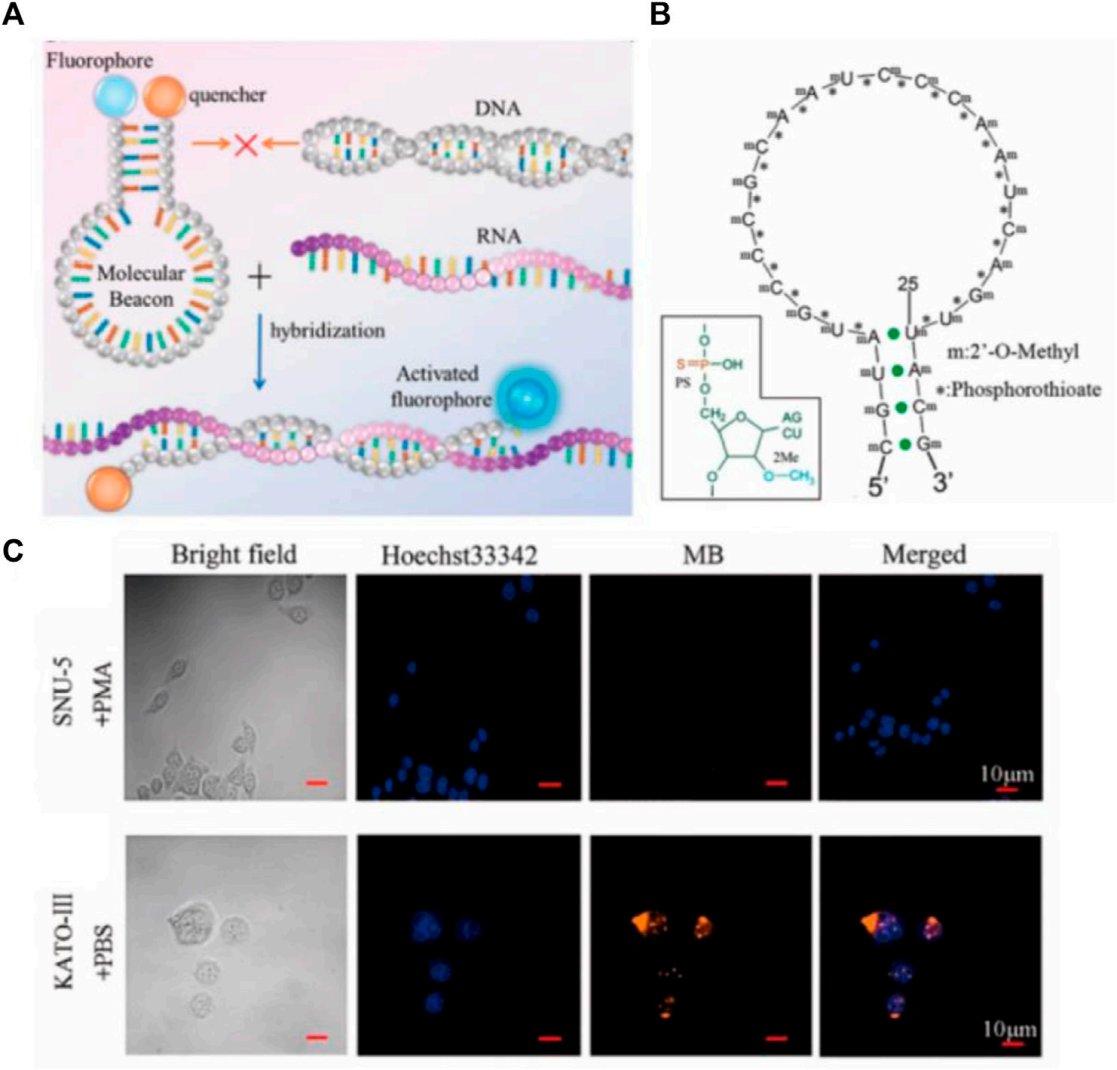
**(A)** Working mechanism of MB hybridization reaction with target. **(B)** Schematic diagram of MB modified with thiophosphoric acid and 2′-o-methyl. **(C)** *In situ* imaging of CLDN18.2 RNA in cells by MB (Fan et al., 2021). Copyright 2021 American Chemical Society.

Molecular Beacon (MB) is a new type of DNA fluorescent probe developed by Tyagi and Kramer in 1996 (Figure 10A) (Tyagi and Kramer, 1996), which is a hairpin structure formed by DNA, composed of stem and loop. Usually, the loop portion of the MB is the complementary sequence of the detection target for specific recognition, and the stem of the DNA mainly plays a fixed role to ensure the stability of the MB. In the absence of the target, the MB is present in a hairpin conformation, at which point the fluorophore and the quenching group are close to each other, and the fluorescence of the fluorophore is quenched due to the action of fluorescence resonance energy transfer (FRET). When the MB binds to the target, the loop portion of the MB and target form a more stable double chain than the stem, which leads to the structural recombination of the MB hairpin. At this time, the loop is opened, whereas the stem quenching group and the fluorophore are separated, then the fluorescence is restored (Tan et al., 2004). MB was first exploited for the detection of small biological molecules of RNA *in vitro*, and then it was used in cells to realize the *in situ* detection of biological small molecules gradually. It is difficult for MB alone to enter the cell, resulting from absorbance on the cell surface. Consequently, through microinjection or transfection reagent, the researchers can make MB enter the cells smoothly to realize the localization and quantitative detection of small biological molecules in the cells. Gewirtz et al. used microinjection technology to inject MB into cells to realize *in situ* analysis of intracellular biological small molecules (Sokol et al., 1998). The difficult operation of microinjection technology requires professional operation, limiting its universal practicality. Li’s group used Lipofectamine^®^ 2000 to transfect MB into cells for detection *in situ* (Li and Wong, 2010). With transfection reagent accessible to acquire, Li’s method greatly reduces the complexity of the operation and improves universal practicality compared with microinjection. Although MB provides a powerful tool for detection and cell imaging, non-specific separation of fluorophores and quenchers often leads to false positive signals due to the degradation of DNase I (Chen et al., 2007). To overcome the generation of these false positive signals, the researchers improved MB to ensure its stable presence inside cells. Seo et al. developed a novel MB that uses spherical graphite nanoparticles as fluorescent quenchers, and the presence of spherical graphite nanoparticles enhances the biological stability of the MB and the ability to transfect cells, enabling real-time detection and quantification of surviving mRNA in MCF-7 cell (Piao et al., 2012). To improve the efficiency of probe entry into the cell and the stability of the intracellular environment, Shen’s group optimized CLDN18.2 MB by applying phosphorothioate and 2′-O-methyl methods to achieve *in situ* intracellular detection of CLDN18.2 RNA (Figure 2) (Fan et al., 2021). These improvements greatly enhanced the ability of MB to enter cells and the stability of the intracellular environment, making molecular beacons a reality for accurate *in situ* detection of tumor-associated intracellular RNA.

Other than conventional MB, dual-color MB has also attracted more and more attention. It is universally acknowledged that the proportion recognition of the human eye to two mixed colors is much higher than the recognition of a single color. A single color can only be distinguished by the brightness of the color, while the ratiometric color of two mixed colors can be recognized directly by the color difference (Jiang C. et al., 2018; Yang et al., 2019). Fluorescence resonance energy transfer (FRET) is the process by which an excited donor fluorophore transfers nonradiative energy to the chromogenic fluorophore of the acceptor, and consequently, the characteristic fluorescence emission of the acceptor occurs at the excitation wavelength of the donor (Roy et al., 2008). FRET is useful in stably improving signal-to-background ratio as well as effectively avoiding the generation of false positive signals (Tsourkas et al., 2003; Martí et al., 2007; Chen et al., 2009). Therefore, some dual-color ratiometric MB based on FRET were designed. Bao’s group report a method for detecting K-ras mRNA and surviving mRNA with FRET-based dual-MBs. At this point, two conventional molecular beacons are bound on a single target, when the two fluorophores are quite close to each other, and the FRET occurs to form a dual-color ratiometric detection (Santangelo et al., 2004). Compared with the traditional monochrome MB, FRET-based dual-color MB not only realizes ratiometric fluorescence detection but also avoids false positive signals to a great extent. Our group designed a dual-colored ratiometric fluorescent oligonucleotide probe to detect telomerase RNA (hTR) in cell lysis, based on molecular beacons (Figure 10B) (Ning et al., 2017). A long DNA sequence (modified with a quenching group) and a dual-color MB (modified with FAM and TAMRA) are combined to form the probe. In the presence of the target, the long DNA sequence combined with the target to form a more stable double strand, and the MB returned to the hairpin structure, forming a ratiometric fluorescence detection model. The probe realized the visual evaluation of hTR level in cell lysate and the discrimination of cancer cells and normal cells. Modifying the probe to enter the cell efficiently and stabilizing it in the cell environment were necessary to realize *in situ* ratiometric detection, which has a good application prospect to form a visual assessment of lysate and the integration of ratiometric detection *in situ*.

### Electrochemiluminescence

**FIGURE 3:**
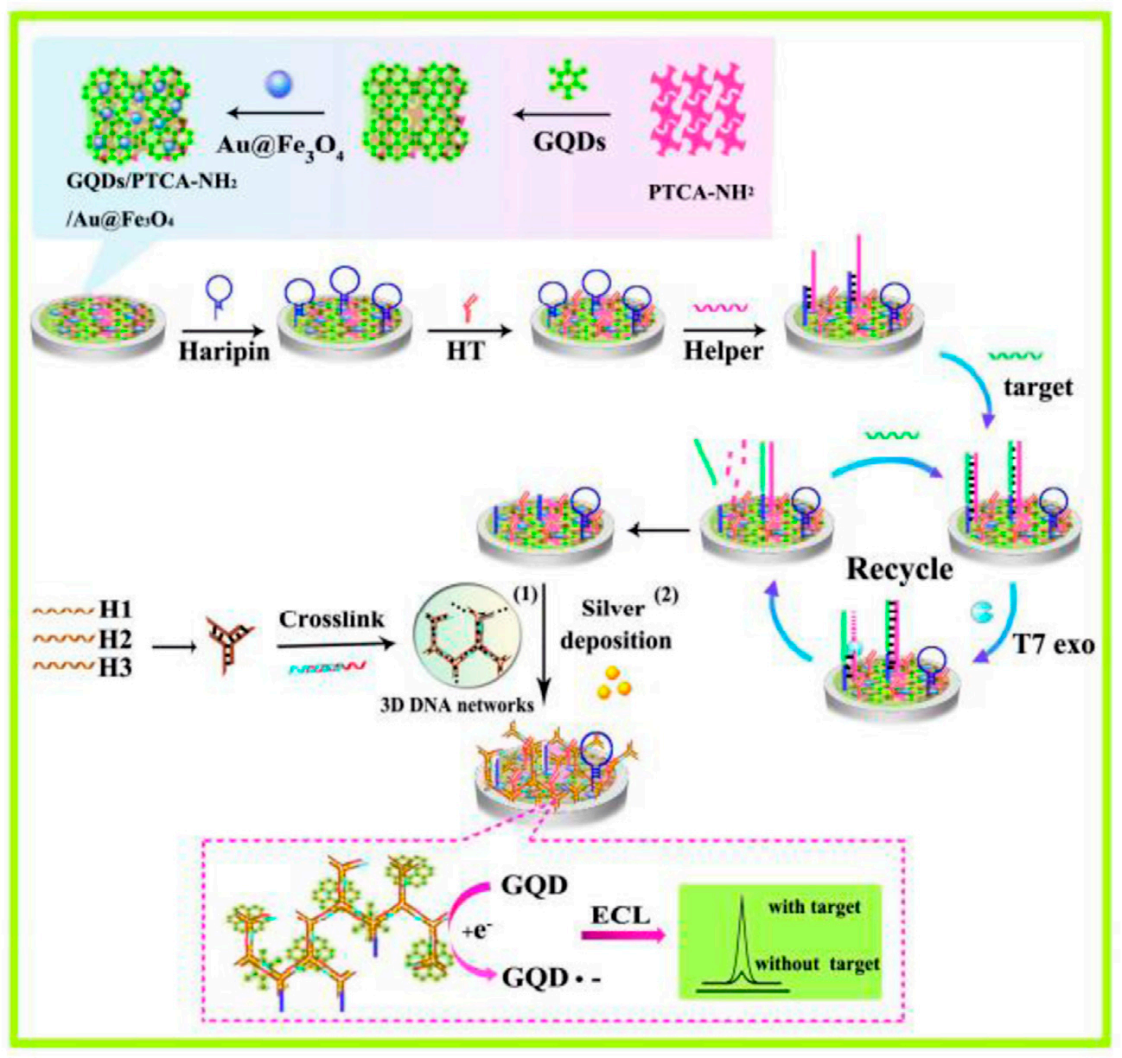
Working mechanism of electrochemiluminescent graphene quantum dots sensing platform for the detection of miRNA-21 (Zhang et al., 2015). Copyright 2015 American Chemical Society.

The development of nanomaterial-based nucleic acid detection systems has taken fluorescent probes to a new level. In certain cases, it may have favorable characteristics which make it attractive for a wide range of applications. At present, the detection of biological small molecules faces many challenges, such as low content and more interference, thus an electrochemical-based detection method was established. Electrochemiluminescence (ECL) has garnered rising interest in the detection of DNA and miRNA due to the unique properties of minimal background signal, easy optical setup, and high sensitivity. (Liang et al., 2017). Years of widespread use of commercial ECL DNA probe assays in a variety of applications has shifted the research focus to the development of miRNA detection, involving the production of reductants by electrochemical oxidation in the presence of both the luminophore and corectant. In a typical ECL miRNA analysis system, gene probes were initially mounted on an electrode surface including a component with a target-complementary sequence. In the presence of target miRNA, miRNA can hybridize with the complementary sequence. The chosen luminophore should be able to be collected to the working electrode, then the luminophore releases electrons and performs an oxidation reaction when a particular voltage energy is given to the working electrode. Simultaneously, the corectant upon electrode surface releases electrons and performs an oxidation process to create the cationic radical corectant, and removes a proton rapidly and spontaneously to generate reducing corectant. The strong oxidizing luminophore and the strong reducing radical corectant undergo a redox reaction. Oxidizing luminophore is reduced to excited state, then it decays *via* a fluorescence mechanism and releases energy by emitting a photon. In the meantime, the ground state luminophore is restored and prepared for the subsequent cycle. (Hu and Xu, 2010). There have been a variety of ECL systems reported, such as the classical [Ru (bpy)_3_]^2+^/TPrA system and Ag NCs/S_2_O_8_^2−^ system. With rational design of “luminophore-corectant” ECL system and probe, it processes great potential for future biomedical applications. Nevertheless, the time-consuming signal amplification method requires further modification (Chen et al., 2016). Chai’s group reported an electrochemiluminescent graphene quantum dots sensing platform for detecting miRNA-21 (Figure 3) (Zhang et al., 2015). Chen et al. reported a novel coreactant-free electrochemiluminescence biosensor for detecting miRNA-21 (Figure 10C) (Zhao et al., 2020). The detection limits of miRNA-21 in these two ECL-based methods are one fM and 33 aM, which highly improves the sensitivity and reduces the detection limit. This provides a new idea to detect the trace amount of some small biological molecules in cells. Nevertheless, the electrochemical detection method is generally only used for *in vitro* detection, and few reports are applied to the *in situ* detection of small biological molecules in cells.

### AuNP-based nanoprobes

Gold nanoparticles (AuNP) was synthesized by Michael Faraday more than 150 years ago, possessing a long history in the field of chemical research (Daniel and Astruc, 2004; Hayat, 1989). AuNP with diameters in the range of 5–250 nm can be synthesized using the methods of (Frens., 1973) as well as Enustun and Turkevich (Enustun B V, 1963), and the synthesized AuNPs have good homogeneity and dispersibility. The above AuNP synthesis method with precisely controlled size provides the basis for the application of gold nanoprobes in cell biology in recent years. AuNP has unique physical and chemical properties: 1) Excellent fluorescence quenching ability. When the distance between AuNP and fluorescein gets close, resonance energy transfer takes place to cause fluorescence to be quenched. Dubertret et al. (2001) demonstrated that the quenching ability of AuNP for fluorescent dyes emitted near infrared is better than that of DABCYL. 2) Good fluorescence quenching distance. Dulkeith’s group demonstrated that AuNP has a good quenching effect on fluorescent dye Cy5 in the range of 2.2 nm–16.2 nm (Dulkeith et al., 2005). 3) Entering cells through endocytosis does not require the help of transfection reagents. Mrikin’s group demonstrated that DNA functionalized AuNP targets class A scavenger receptors and endocytosis *via* a lipid-raft–dependent, caveolae-mediated pathway (Choi et al., 2013). 4) Outstanding stability. DNA was connected to the surface of AuNP by gold sulfur bond and formed a dense shell on the surface of AuNP. Mrikin’s group demonstrated that DNA functionalized AuNP can remain stable in the environment of DNase I (Seferos et al., 2009). 5) Low cytotoxicity. Mirkin et al. observed DNA functionalized AuNP in cell antisense experiments with only slight toxicity (Rosi et al., 2006). 6) Strong specificity. DNA functionalized AuNP reduces fluorescence reporting by at least 50% even when reacting with a single mismatched target. DNA functionalized AuNP can be stable in the physiological environment of metal ions and glutathione (GSH), which further illustrates its strong specificity (Zheng et al., 2009).

**FIGURE 4:**
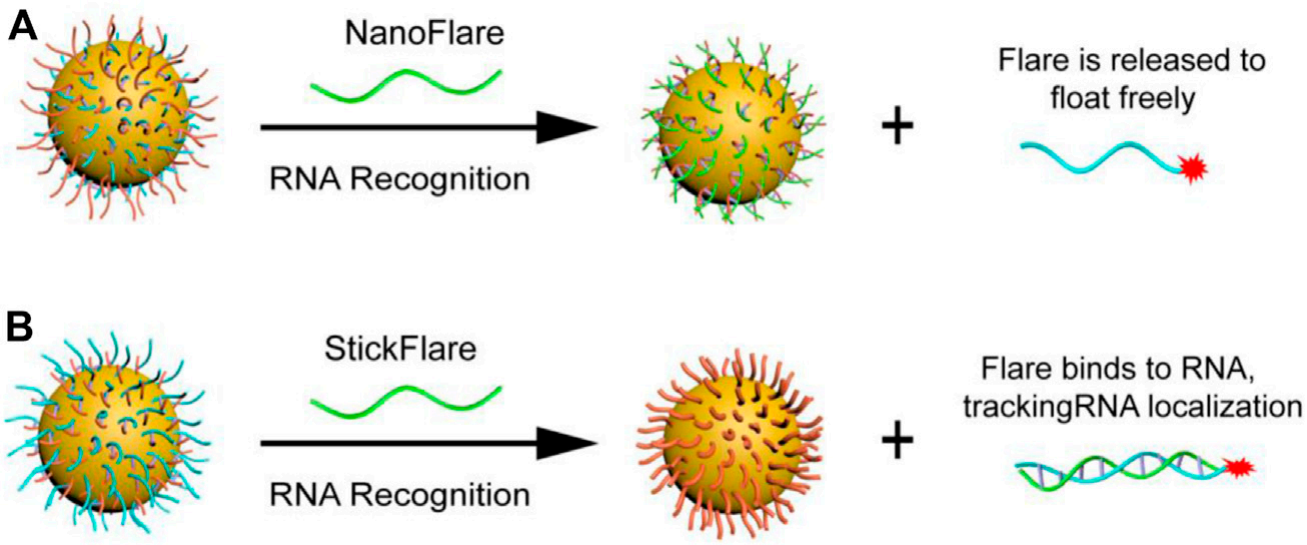
**(A)** NanoFlare and **(B)** StickFlare (Zhao et al., 2021) for tumor-related RNA detection. Copyright 2021 The Royal Society of Chemistry.

Mrikin’s group proposed nanoFlare in 2007 (Figure 4A) (Seferos et al., 2007), which fully hybridizes the recognition sequence modified by Cy5 and the long DNA sequence modified by the sulfhydryl group, and connects it to the surface of AuNP through the gold sulfur bond to form nanoFlare. Due to the quenching effect of AuNP, the fluorescence of Cy5 is quenched. When the target is present, the long DNA sequence forms a more stable double strand with the target, and the recognition sequence modified by Cy5 falls off the surface of AuNP. This process is accompanied by fluorescence recovery, resulting in a specific detection. Tang et al. constructed a triple-fluorescent nanoFlare on this basis, which can simultaneously achieve the detection of three mRNAs (c-myc mRNA, TK1 mRNA, and GalNAc-T mRNA) *in situ* (Li N. et al., 2012). Compared to traditional single-fluorescent nanoFolare, triple-fluorescent nanoFlare can simultaneously detect three tumor-related RNAs and effectively prevent false-positive signals. In 2015, Mrikin’s group improved nanoFlare and designed stickyFlares (Briley et al., 2015). A short DNA sequence with modified sulfhydryl group is hybridized with a long Cy5 recognition sequence to form a double strand, which is then connected to the surface of AuNP to form stickyFlares. When the target is present, the Cy5 recognition sequence and the target form a more stable double strand and fall off the AuNP, forming a specific *in situ* detection. Our group also designed different stickyFlares to detect telomerase RNA (hTR) and tumor suppressor gene p21 mRNA in cells, respectively (Figure 4B) (Wu et al., 2018; Zhao et al., 2021). The main difference between stickyFlares and nanoFlare is whether it can determine and track the spatial distribution of RNA in cells. For nanoFlare, when RNA binds to nanoFlare, the fluorescent recognition sequence falls off from the surface of AuNP. At this time, the RNA is connected to AuNP, therefore, nanoFlare cannot determine and track the spatial distribution of RNA in cells. Nevertheless, when stickyFlares bind to the RNA, the fluorescence recognition sequence and RNA form a stable double strand, which falls off from AuNP and produces fluorescence. Therefore, stickyFlares can enable the determination and tracking of the spatial distribution of RNA in cells.

**FIGURE 5:**
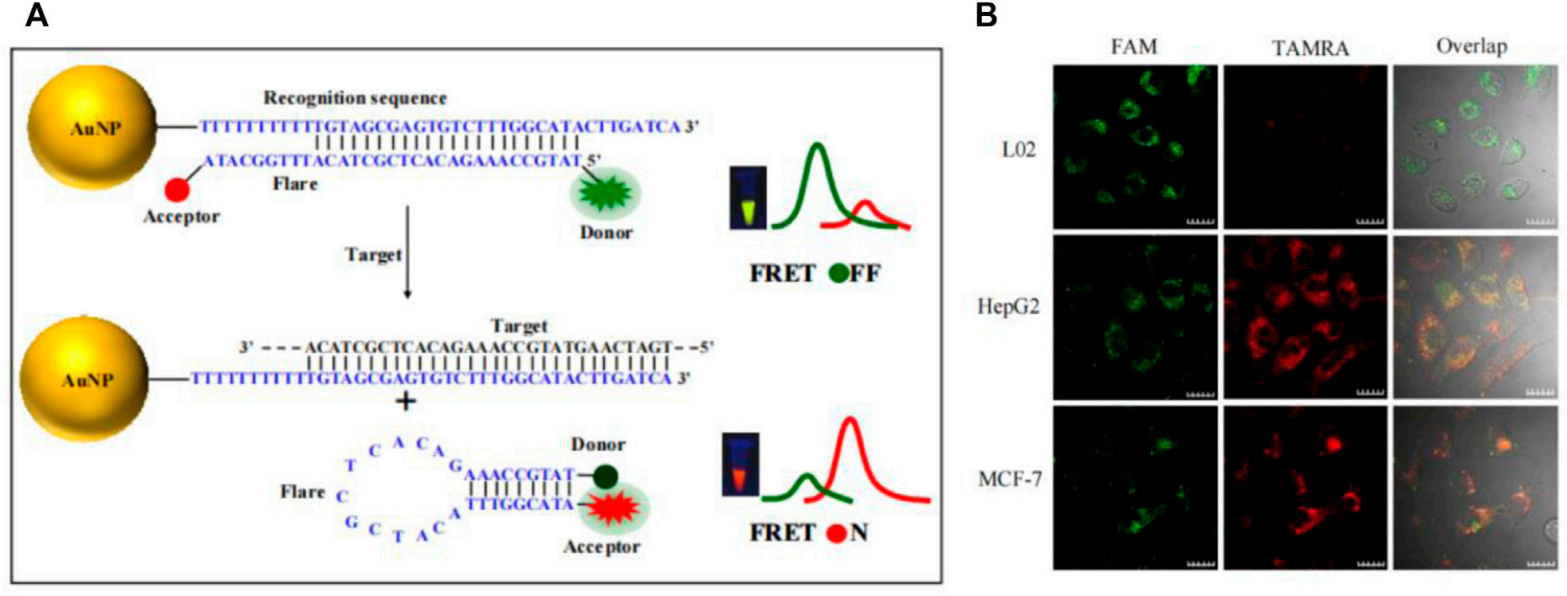
**(A)** Working mechanism of TK1 mRNA detection based on FRET nanoFlare. **(B)** *In situ* ratiometric imaging of TK1 mRNA in HepG2 cell, L02 cell and MCF-7 cell by FRET nanoFlare (Yang et al., 2015a). Copyright 2015 American Chemical Society.

Aside from stickyFlares and nanoFlare, a number of AuNP-based molecular beacon probes have also been designed. Qiao et al. developed an AuNP-based dual-color molecular beacon probe that simultaneously performs *in situ* detection of surviving mRNA and cyclin D1 mRNA in breast cancer cells (Figure 10D) (Qiao et al., 2011). Tang’s group designed an AuNP-based four-fluorescent molecular beacon that simultaneously detects four mRNAs (TK1 mRNA, surviving mRNA, C-myc mRNA, and GalNAc-T mRNA) in cells (Pan et al., 2013). Both detection methods provide more reliable information for early diagnosis of cancer by avoiding false positives, in which single tumor-related RNA was detected. Nucleic acid functionalized AuNP probes have been further improved by researchers since the development of Mirkin’s team, but the detection limits of nucleic acid functionalized AuNP probes has always been high (generally 1–10 nM), resulting in huge challenges in the detection of trace amount of biomolecules in cells. Jiang et al. developed a novel electrostatically assembled nucleic acid nanostructure based on AuNP and applied the hybridization chain reaction (HCR) amplification technique to the detection of intracellular surviving mRNA (Wu et al., 2015). This kind of probe can detect mRNA at the pM level, which significantly improves the sensitivity of detection and successfully overcomes the problem of detecting trace amounts of small molecules in cells. To evaluate the amount of intracellular mRNA by ratiometric fluorescence signaling and minimize the effect of system fluctuations, some AuNP-based dual-color ratiometric probes have also emerged. Wang et al. constructed a molecular beacon-based FRET nanoFlare that realized the ratiometric fluorescence analysis in cells through the resonance energy transfer of fluorescein FAM and TAMRA (Figure 5) (Yang et al., 2015b). Compared to traditional monochromatic nanoflares, FRET-based dual-color ratiometric nanoflare avoids chemical interferences (such as DNase I and GSH) and false positive signals generated by thermodynamic fluctuations. Xie et al. combined AuNP with tetrahedral DNA nanostructures to structure a novel nanosensor for fluorescence resonance energy transfer, enabling *in situ* ratiometric fluorescence analysis of miRNA-21 in cells (Bai et al., 2018). Some AuNP-based functionalized nucleic acid probes were systematically introduced above. These AuNP-based functionalized nucleic acid probes have been continuously improved, and the diversification, sensitivity and specificity of the probes have been greatly developed, which will have better application prospects in the future.

### GO-based nanoprobes

**FIGURE 6:**
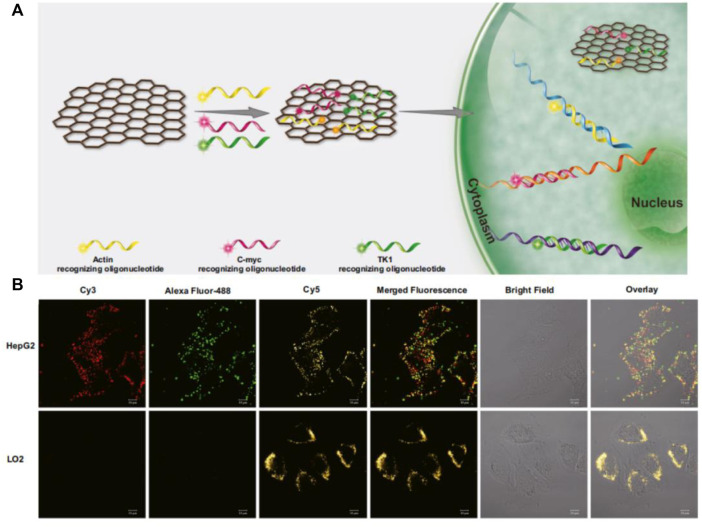
**(A)** Working mechanism of GO-based nanoprobe detection. **(B)** The GO-based nanoprobe simultaneously imaging detection of three mRNAs in cells. (Jiang H. et al., 2018). Copyright 2018 Springer-Verlag GmbH Austria.

The question of the existence of graphene has puzzled researchers for many years (Wallace, 1947). Until 2004, Giem’s group invented a technique for stripping single-layer graphite, known as graphene, and the study of graphene was transformed from theory to reality (Novoselov et al., 2004). Based on the stripping method of graphene oxide (GO) has broad prospects in large-scale production and application, such as composites, gas sensors, supercapacitors, etc, (Park et al., 2009). Since the mid-20th century, graphene oxide (GO) has usually been synthesized by Hummers’ method (Hummers and Offeman., 1958), and then a variety of synthesis methods have been developed on this basis (Daniela et al., 2010). Compared with graphene, the surface of GO has many oxygen-containing functional groups, such as the epoxy group, carboxyl group, and hydroxyl group. As a common nanomaterial, GO is widely used by researchers, due to its unique structure, mechanical and thermal conductivity, high electron mobility, large surface area, good biocompatibility, and strong design (Takada, 2019; Hu et al., 2021). It can be seen in the detection of metal ions, small organic molecules, and biomolecules (Li et al., 2013; Huang et al., 2015; Qu et al., 2016). Here, we focused on the application of GO in the detection of tumor-related RNA. As a flaky structure with a positive charge on its surface, GO was utilized in adsorbing single fluorescent stranded DNA on the surface through the interaction of the charge and effectively quenching fluorescence by stacking π-π (Xu et al., 2019). When the target binds with the single fluorescent stranded DNA to form a double strand and then falls off from the surface of GO to produce fluorescence. Fan et al. designed a GO-based molecular beacon probe, which significantly reduced the background fluorescence and increased signal-to-background ratio, sensitivity and thermal stability compared with a single molecular beacon (Li et al., 2010). Ling’s group developed a multiplexed graphene oxide (GO) fluorescent nanoprobe, which can detect three kinds of mRNA (C-myc mRNA, Actin mRNA, and TK1 mRNA) in cells at the same time (Figure 6) (Jiang H. et al., 2018). The probe uses Actin mRNA as an internal control to discharge false positive signals due to different cells’ different intakes of nanoprobes. Moreover, GO-based nanoprobe is capable of distinguishing between cancer cells and normal cells, facilitating the analysis of cancer progression. Although GO has been used for *in situ* detection of intracellular tumor-related RNAs, its low quenching ability and genotoxicity to cells and tissues directly determine its limited range of applications *in vivo*. Hence, the researchers successfully developed a low-toxicity, high-quenching reduced graphene oxide (rGO) probe for the detection of biological small molecules in cells. Huang’s group compared the quenching efficiency of GO and rGO for the three fluorescent dyes. The results showed that the quenching ability of rGO was higher than that of GO, and the improvement of quenching ability also greatly reduced background fluorescence (Kim et al., 2010). Wang’s group reported an rGO-based biosensor that simultaneously detects and monitors the two tumor suppressor genes p21 mRNA and p53 mRNA in cells, which is of great significance in the early diagnosis and prognosis evaluation of cancer (Figure 10E) (Fan et al., 2019). Although rGO nanoprobes showed significant advantages over ultra-low toxicity, their detection limits are as unobtrusive as those of GO nanoprobes, and thus it is crucial to increase the sensitivity and reduce the detection limit of it. Liu et al. reported a fluorescence biosensor based on rGO coupling with duplex-specific nuclease signal amplification (DSNSA), which greatly builds up the sensitivity with the help of DSN, reducing the detection limit of the probe (100 fM) to achieve *in situ* detection of VEGF mRNA inside cells (Dang et al., 2019). In conclusion, in the nucleic acid detection system of GO and its derivatives, GO nanomaterials were firstly used to adsorb probes with covalent fluorophores, resulting in fluorescence quenching. In the presence of target, the probe binds with the target and falls off the surface of the GO and its derivatives, and produces a fluorescent signal. This method has the advantages of simplicity, low toxicity, high signal strength and high signal-to-noise ratio, which holds great promise in the application.

### MnO_2_-based nanoprobes

**FIGURE 7:**
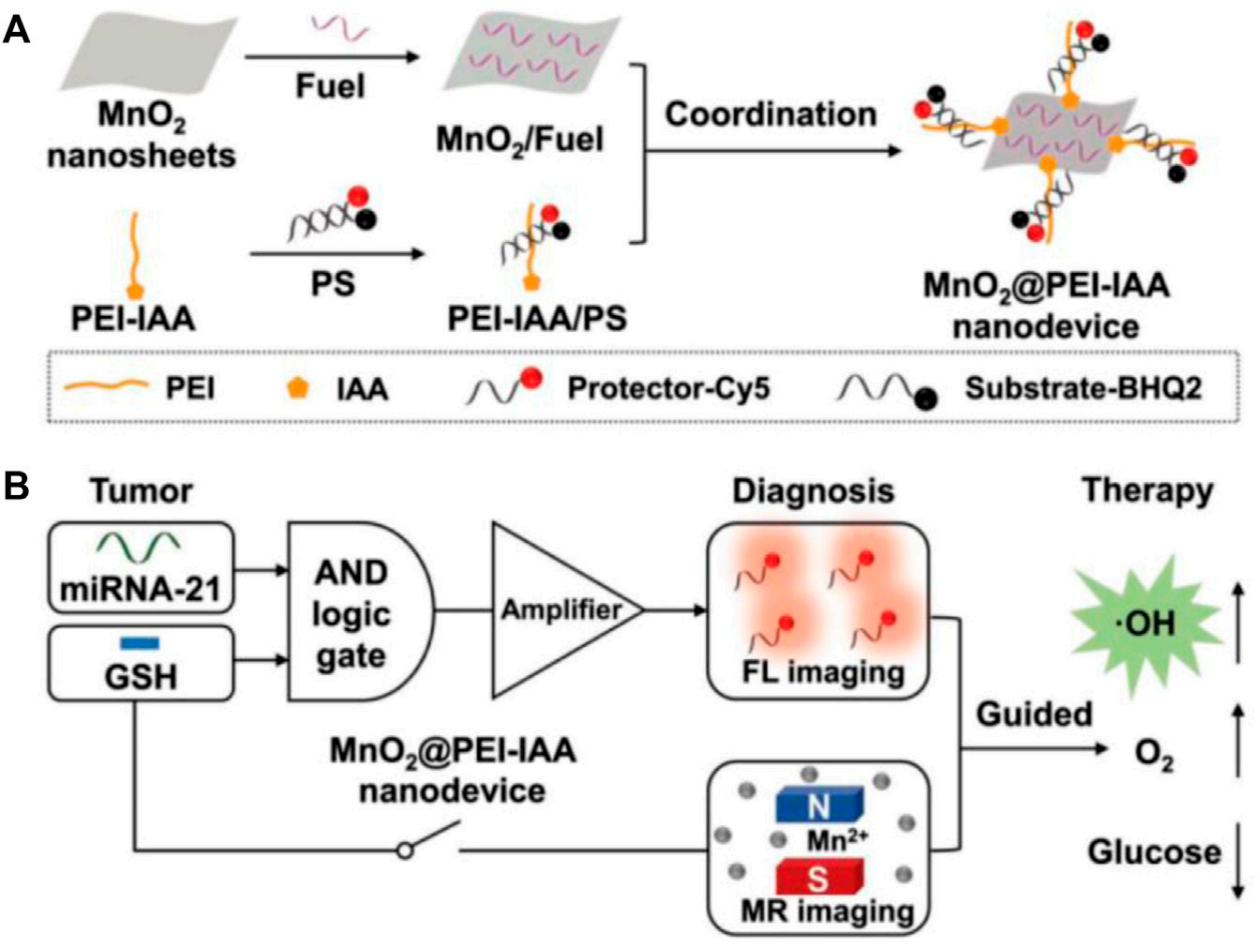
**(A)** Schematic diagram of the construction of MnO_2_@PEI-IAA nanodevice. **(B)** MnO_2_@PEI-IAA is used for cascaded signal-amplified FL imaging of miRNA-21 and MR imaging-guided therapeutics for GSH activation (Yan et al., 2019). Copyright 2019 Wiley.

In recent years, the increasing importance has been attached to MnO_2_ nanosheet for the detection of small biological molecules because of their unique physical and chemical properties: (Zhai et al., 2014; Zhao et al., 2014). 1) MnO_2_ nanosheet is a nanomaterial with a flake structure, which has a wide absorption peak between 300 and 600 nm and has a strong quenching effect on the fluorescence of this range. 2) MnO_2_ nanosheet can adsorb single stranded DNA by synergistic physical adsorption. 3) MnO_2_ nanosheet has good biocompatibility and is used as a vector to carry DNA through the cell membrane into the cell. 4) MnO_2_ nanosheet can be reduced to Mn^2+^ by intracellular glutathione (GSH) (Ou et al., 2018). Thus, MnO_2_ nanosheet can be used as a vector to carry a DNA probe into the cell to complete the detection of intracellular small molecules. Wang’s group proposed a novel MnO_2_-based biosensing platform for detecting DNA hybridization and identifying biological small molecule interactions in solution (He et al., 2014). To reduce the effects from background fluorescence, wang’s group designed a dual-color MnO_2_ probe based on FRET to achieve fluorescence ratio imaging of TK1 mRNAs in cells and distinguish between normal and cancer cells (Figure 10G) (Ou et al., 2017). Highly sensitive mRNA detection is critical to better understand the role of mRNA in cells and further validate its function in clinical diagnosis. Therefore, it is crucial to design a more sensitive probe for the detection and monitoring of intracellular tumor-related mRNA sensitivity. Tian et al. designed a GSH-gated DNA nanodevice for miRNA-21 and magnetic resonance (MR) imaging with the help of MnO_2_ (Figure 7) (Yan et al., 2019). Compared to ordinary probes, such probes can not only perform *in situ* detection of tumor-related RNA but also guide chemodynamic therapy (CDT). The main design principle of MnO_2_-based nucleic acid probes is that the single-stranded DNA sequence with fluorescence adsorbs on MnO_2_ and fluorescence is quenched. In the presence of the target, the target binds to the single stranded DNA sequence modified by fluorescent to form a more stable double strand, which falls off from MnO_2_ and recovers fluorescence, resulting in a specific detection. This detection method is similar to the principle of the above-mentioned GO functionalized nucleic acid probe, both of which belong to flake nanoprobes. However, such nanoprobes are all sheet-like structures, which may be difficult to enter the cell, and take a long time to complete the detection in cells.

### SiO_2_-based nanoprobes

**FIGURE 8:**
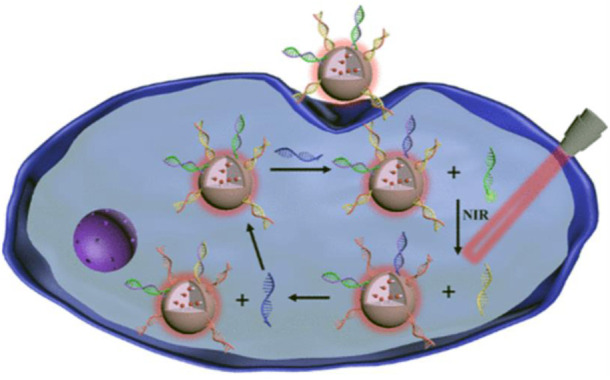
Working mechanism of Ru-SiO_2_@ polydopamine nanoplatform for MicroRNA detection in cells (Deng et al., 2021). Copyright 2021 American Chemical Society.

The size of silicon dioxide (SiO_2_) is generally in the range of 50–200 nm. Due to its high drug loading and inert structure, it allows the encapsulated drugs to remain high stability in cells, which has captured much more attention (Li Z. et al., 2012; Graybill and Bailey, 2016; Pan et al., 2016). However, a small number of researchers have developed SiO_2_-based fluorescent probes for the detection of tumor-related RNAs in living cells. Wu’s group developed a NIR-assisted signal amplification based on Ru-SiO_2_@ polydopamine nanoplatform that realizes the ratiometric imaging of miRNA in cells (Figure 8) (Deng et al., 2021). With its good specificity, stability, and low toxicity, this probe avoids false-positive signals caused by DNase I, opening up a new path for sensitive detection of trace amounts of mRNA. The surface of SiO_2_ contains many pores, which provides a good platform for drug loading. Therefore, the SiO_2_ probe can fully utilize the characteristics of SiO_2_ by integrating detection and drug loading. Yuan et al. (2017) developed the mesoporous silica nanoquencher that not only enable the detection and fluorescence imaging of surviving mRNA in cells but also release loaded drugs based on the content of surviving mRNA in tumor cells to complete chemotherapy (Figure 10F). This integrated detection and treatment probe can greatly reduce the toxicity of the drug and make the drug have better targeting, which can improve the therapeutic effect and reduce the damage to normal cells, holding great prospect in the integration of detection and treatment for tumor-related RNA. However, there are relatively few studies on the application of SiO_2_ nanoprobes and the *in situ* detection of small biomolecules in cells because SiO_2_ itself is a good drug carrier, which lead to pay more attention to the drug loading performance of SiO_2_. Therefore, the study of SiO_2_-based nanoprobes still face huge challenges in the *in situ* detection of biomolecules in the future, hoping that more probes integrated detection and treatment can be developed to monitor the therapeutic effect of drugs in real time.

### AuNR-based nanoprobes

**FIGURE 9:**
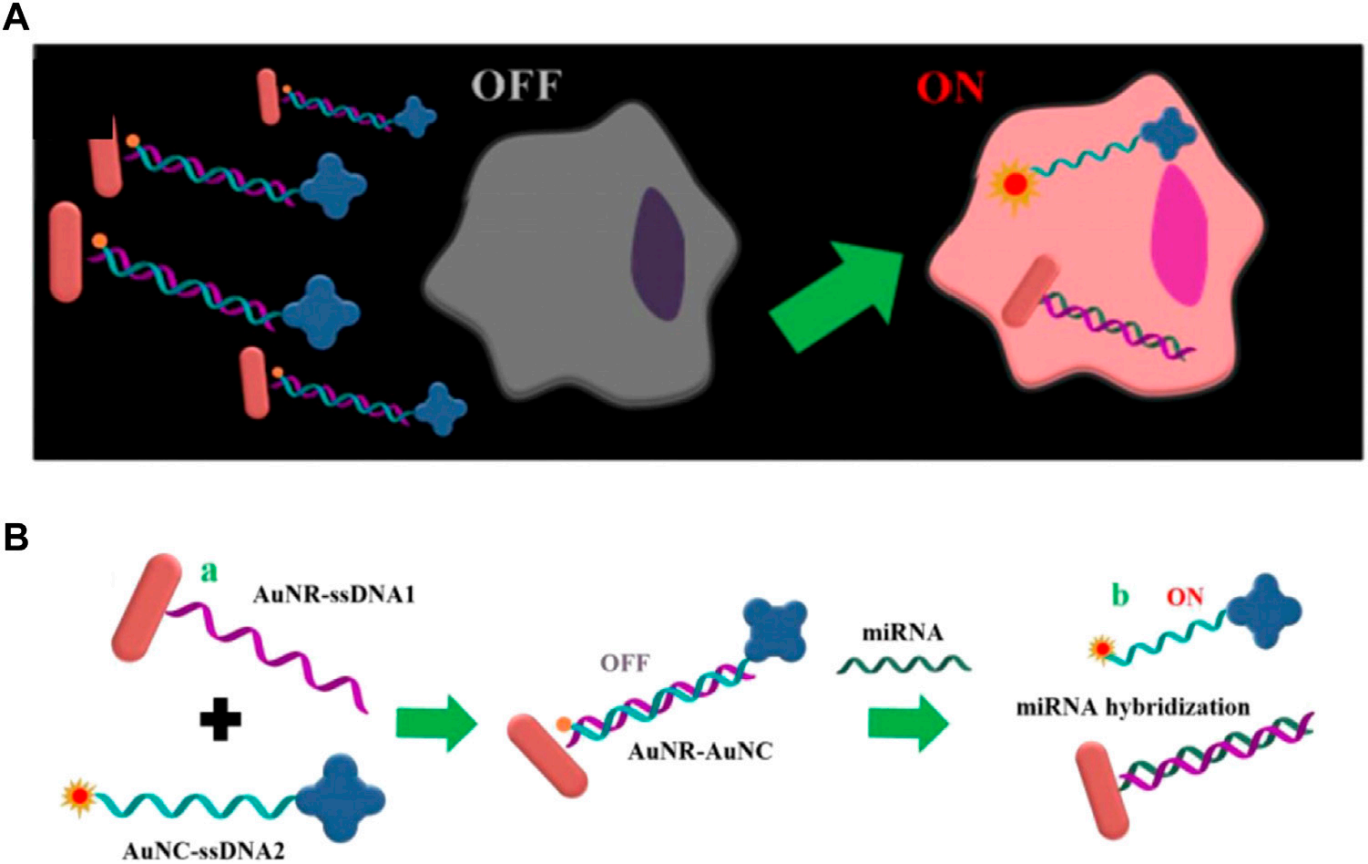
**(A)** Schematic illustrations of “OFF-enhanced ON” fluorescent switch system for specific detection of miR-21 in cancer cells. **(B)** Schematic illustrations of probe assembly and detection (Sun et al., 2018).

**FIGURE 10:**
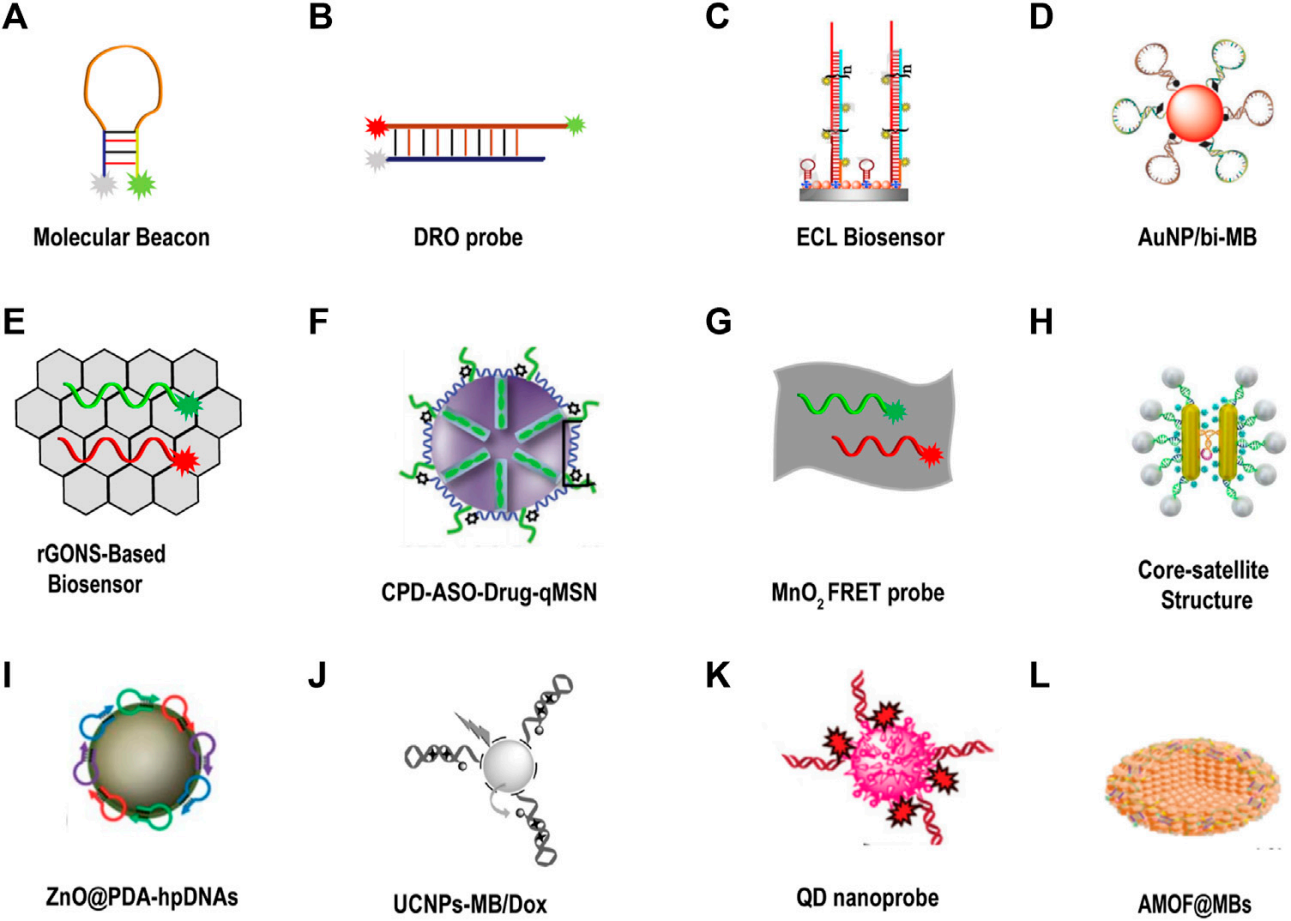
DNA fluorescent probe for tumor-related imaging in living cells. **(A)** Molecular beacon (Tyagi and Kramer, 1996). **(B)** Dual-colored ratiometric fluorescent oligonucleotide probe (DRO) (Ning et al., 2017). **(C)** Electrochemiluminescence (ECL) biosensor (Zhao et al., 2020). Copyright 2020 Elsevier. **(D)** AuNP was assembled with a bi-molecular beacon (bi-MB), and termed AuNP/bi-MB (Qiao et al., 2011). Copyright 2011 Wiley. **(E)** rGONS-based biosensor (Fan et al., 2019). **(F)** CPD-ASO-Drug-qMSN (Yuan et al., 2017). Copyright 2017 Wiley. **(G)** MnO_2_ nanosheet mediated “DD–A″ FRET binary probes (Ou et al., 2017). **(H)** Core-satellite structure (Ma et al., 2017). Copyright 2018 American Chemical Society. **(I)** ZnO@PDA-hpDNAs (He et al., 2017). Copyright 2017 The Royal Society of Chemistry. **(J)** UCNPs-MB/Dox (Ding et al., 2016). Copyright 2016 Wiley. **(K)** QD nanoprobe (Lin et al., 2021). Copyright 2020 The Royal Society of Chemistry. **(L)** AMOF@MB (Liu et al., 2019). Copyright 2019 The Royal Society of Chemistry.

Gold nanorods (AuNR) is a kind of gold nanomaterials ranging in size from a few nanometers to hundreds of nanometers, which possesses excellent properties such as surface plasma resonance and thermogenesis performance, consequently, it is widely used in biosensors and photothermal treatment of tumors (He et al., 2008; Kennedy et al., 2011; Li et al., 2015). Owing to the excellent improvement of the sensitivity of the probe through the surface plasmon resonance, AuNR has been successfully applied to *in vivo* imaging detection, which will be further introduced later. Xu’s group reported DNA-programmed AuNR dimer-UCNP core-satellite assemblies as SERS- and luminescence-based probes, which simultaneously detect dual targets for telomerase and miR-21 in living cells (Figure 10H) (Ma et al., 2017). This dual-target probe allows to detect both tumor biomarkers at the gene level and protein level at the same time, which is of great significance for the early detection of tumors. Sun et al. developed a gold nano-conjugates-based “FRET-SEF” probe for *in situ* detection of miR-21 in cells (Figure 9) (Sun et al., 2018). The detection limit of this probe is as low as aM level, which largely improves the sensitivity of the probe and opens up a new path for the detection of trace amounts of mRNA in cells.

### Other nanoprobes

In addition to several nanoprobes mentioned above, many nanomaterials have been successfully utilized for the detection of tumor-related RNA. Li et al. developed a ZnO@polydopamine-nucleic acid nanosystem for the detection of surviving mRNA (Figure 10I) (He et al., 2017). Xing’s group developed an upconversion nanobeacon for the ratio fluorescence detection of TK1 mRNA in cells and the treatment of tumor cells with drug-loading drugs (Dox) (Figure 10J) (Ding et al., 2016). Zhao et al. designed a multifunctional nanoprobe based on biomass quantum dots (BQDs) for cell imaging of surviving mRNAs and photodynamic therapy for tumor cells (Figure 10K) (Lin et al., 2021). Yang et al. developed an MB probe based on azore-ductase-responsive functional metal–organic frameworks (AMOFs) for the detection of intracellular VEGF mRNA (Figure 10L) (Liu et al., 2019). With the help of different nanomaterials, a variety of probes can be designed. After decades of development, nanoprobes have overcome many difficulties in biomolecular diagnosis. However, each nanoprobe has its own advantages and disadvantages, and thus the probes for tumor-related RNA detection still face some challenges.

## Challenges and future outlook

Over the past few decades, more and more researchers in the field of biomolecular diagnostics have used nanomaterials to detect tumor biomarkers. Compared to traditional technologies, nanoprobes can meet the needs such as practicality, high sensitivity, and high selective test. Nevertheless, it still faces numerous challenges, such as the design of the probe, stability, sensitivity, specificity, cell cytotoxicity, fluorescence selection, cell permeability, application *in vivo*, etc. The above problems will be discussed below and a few potential solutions will be proposed.

### Probe design and its stability

DNA-based fluorescent probes form a common platform for biometrics due to the identification of nucleic acid and non-nucleic acid targets, ease of synthesis and chemical modification, ease of interfacing with signal amplification protocols, and inherent biocompatibility (Ebrahimi et al., 2020). Although the design of DNA fluorescent probes is diversified, the main design principles are similar. Whether it is the above-mentioned MB or the sticky-flares and nanoflare composed of AuNP surface-modified nucleic acids, the main principle of its design is that the binding capacity of long DNA to long DNA is greater than that between long DNA and short DNA (Qian et al., 2011; Qian and Winfree, 2011; Rogers and Manoharan, 2015). Therefore, the length of the nucleic acid sequence should be optimized in design process, which not only ensure the good sensitivity of the probe but also consider its stability. The stability of the probe is the premise of all detection. If the probe cannot exist stably, its detection is often accompanied by false positive signals. Firstly, the design of DNA fluorescent probes relies on the base interaction between bases, thus the Tm value of double-stranded DNA is supposed to be studied that it can stably exist in the physiological environment of the living body. Secondly, the probes for *in situ* detection of cells also need to consider whether they can be stably exist under the presence of the DNase I. In addition to the above probes, such as GO and MnO_2_-based probes, the design principle is that these two materials have good adsorption and quenching effects on single fluorescent DNA strands. When the probe and target bind, the DNA forms stable duplexes that fall off the surface of the nanomaterial and the fluorescence recovers. The design of this type of probe is relatively simple, and it mainly considers the amount of nanomaterials and DNA fluorescent strands. If there are too many DNA fluorescent strands, the nanomaterials cannot quench the fluorescence well, which will lead to excessive background fluorescence. If there are too many nanomaterials, there will be too much quenching phenomenon, resulting in that adding a small amount of target cannot restore the fluorescence, thus reducing the sensitivity of the probe. Therefore, various factors should be considered when designing probes to ensure the feasibility of probe design.

### Sensitivity and specificity of the probe

During the design of probe, the key consideration is its specificity to the target, and poor specificity of probe will cause false positive results. After the design completed, it is of necessity to select analytes similar to the target for the control experiment to assure the fine specificity. For example, when the target is mRNA, sequences with a single mismatch, two mismatches, or three mismatches with the target are often used as controls to evaluate the specificity of the probe. In addition to the good specificity of the probe, the sensitivity plays another essential role. With poor sensitivity, it is hard to report the trace amount of the target, which puts constraints on the scope of application of the probe. Therefore, some detection methods of signal amplification have been studied, such as rolling circle amplification (RCA) (Zhang et al., 2014), strand displacement amplification (SDA (Yan et al., 2014), hybridization chain reaction (HCR) (Huang et al., 2014), catalyzed hairpin self-assembly (CHA) (Zheng et al., 2013), etc. The emergence of these techniques has greatly improved the sensitivity of probes to make the detection of trace amount of small molecules possible. The complex intracellular environment may also affect the sensitivity of the probe and the accuracy of the signal. When the probe enters the cell, it may not open in the pre-pattern to generate a signal. The longer the probe is in the cell, the more likely it will be affected, thus it is also critical to increase the detection speed of the probe in the cell, that is, the rate of signal release.

### Cytotoxicity and choice of probe fluorescence

The cytotoxicity of the probe is a prerequisite for whether the probe can be used for cell *in situ* detection. The toxicity of the probe should be guaranteed to be very weak, because it may cause irreversible damage to the cells if it is used *in situ* detection. Even if the toxicity of the probe is very weak, the metabolic problems of the probe should be considered. If all or part of the probe cannot be metabolically discharged from the cells, long-term accumulation may cause further damage to the cells. As a result, while designing, micro nanomaterials are optional carriers, because of easier metabolism and excretion. The potential solution to this problem is to use a probe, that is, less toxic and metabolizable or to use a probe with a lower concentration for a shorter period. When selecting fluorescent dyes, the influence of the intracellular environment on fluorescence should be pondered. For example, some fluorescein may be difficult to exist stably in the low pH environment of lysosome or interact with other molecules in cells to affect the detection results. Therefore, when choosing fluorescein, fluorescein with high fluorescence efficiency and good stability may be more suitable for complex environments in some cells.

### Intake of probes

As a natural barrier, the cell membrane can prevent foreign bodies from entering cells and forms a self-protection system. Single nucleic acid probe, such as MB, is inherently difficult to enter the cell. With being brought into the cell through microinjection (Bao et al., 2009) or transfection reagents (Dalby et al., 2004), the single nucleic acid probe is likely to be degraded by DNase I to produce a false positive signal. Apart from the false positives caused by the degradation of DNase I, these methods of transfection can be plagued by low cell viability and allow to alter the expression level of the analyte of interest, leading to erroneous measurements. The development of nanoprobes has largely solved these problems, such as nucleic acid functionalization AuNP to enter cells through scavenger receptor-mediated endocytosis, and this approach has been demonstrated to have little effect on gene expression (Patel et al., 2010; Choi et al., 2013). The size of the nanoprobe is also closely related to the internalization of the nanoprobe by the cell. Chan et al. exhibited that AuNP with a diameter of 50 nm is most susceptible to internalization by Hela cells (Chithrani et al., 2006). In addition to the size of the probe affecting the internalization of the probe by the cell, the amount of DNA on the surface of the AuNP also affects the internalization process. Mirkin’s group first demonstrated that DNA loading on the surface of AuNP plays an important role in the cell intake of probes. For DNA functionalized AuNP which is loaded with more than 8 pmol cm^−2^, cells may have exceedingly high intake of this (Giljohann et al., 2007). Furthermore, whether there is a deviation from the intake capacity of different cells to the probe is also a question that ought to be considered. Through studying the intake of probes by three different types of cells, our group proved that the intake ability of three different cells for probes is almost the same (Wu et al., 2018). A potential way to improve the intake ability of cells for probes is to modify some ligands or antibodies on the surface of the probe to target receptors on the surface of the cell so that the probe enters the cell through different ways to improve the intake ability of the probe.

### Application of probe *in vivo*

**FIGURE 11:**
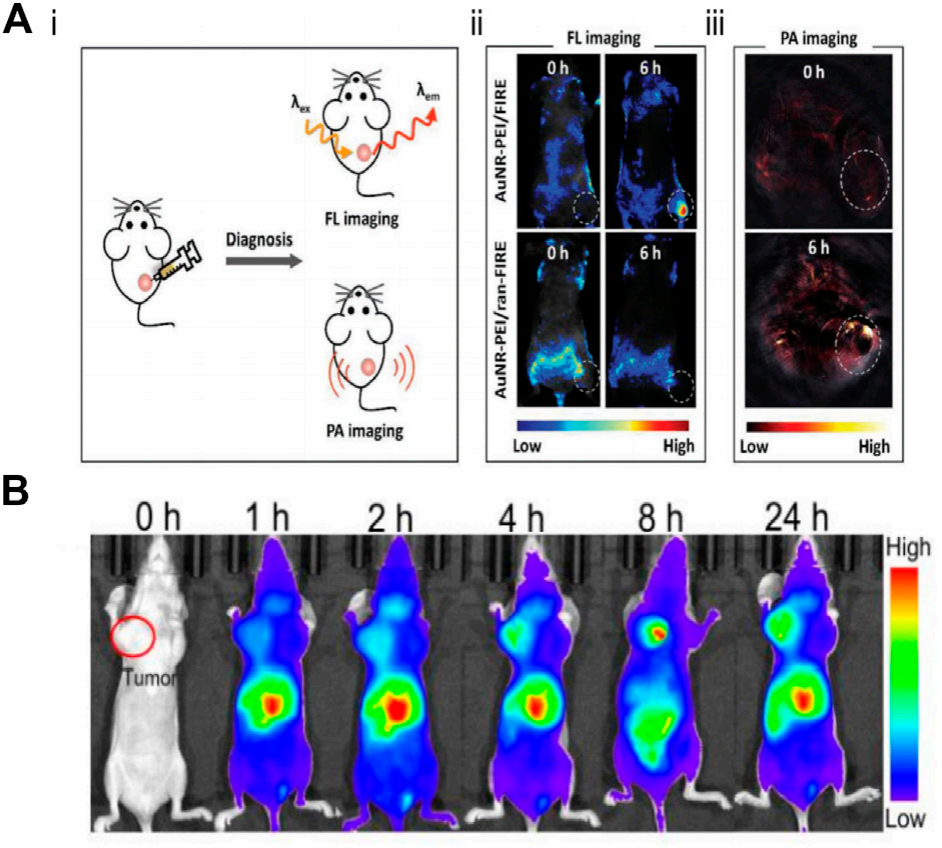
Application of fluorescent nano probe in the detection of tumor related mRNA *in vivo* **(A-i)** Mechanistic diagram of fluorescence (FL) imaging and PA imaging *in vivo*. **(A-ii)** Whole-body FL images of MCF-7 tumor-bearing mice injected with AuNR-PEI/FIRE or AuNR-PEI/ran-FIRE. **(A-iii)** PA image of tumor-bearing mice after injection with AuNR-PEI (Yan et al., 2018). Copyright 2018 Wiley. **(B)** Apt-DNA-Au nanomachines for *in vivo* fluorescence imaging at different times (Yu et al., 2021). Copyright 2020 Wiley.

The application of nanoprobes *in vivo* takes more factors into account than in the application of cells. First of all, after entering the bloodstream through intravenous nanoprobes, it is necessary to successfully avoid the immune system, so as not to be cleared by the immune system before reaching the designated site. Second, the nanoprobe is also required to remain stable during circulation, because the complex environment in the blood may bring about degradation of the probe. Finally, the nanoprobes can accumulate at the target site, resulting in the obvious signal (Ebrahimi et al., 2020). Accordingly, it is a huge challenge to achieve detection *in vivo*. Chen et al. reported the system of AuNR-PEI/FIR, which not only realized the *in vivo* detection of miR-21, but also took into account the photothermal therapy, and realized the integration of *in vivo* detection and solid tumor treatment (Figure 11A) (Yan et al., 2018). In addition, Zhu’s group used AuNP to achieve the detection of TK1 mRNA *in vivo* and realized the combination of photothermal therapy (PTT), photodynamic therapy (PDT), chemotherapy and gene therapy for solid tumors, which made full use of the physical properties of AuNP, strong modifiability, and fine thermal conductivity (Figure 11B) (Yu et al., 2021). Although this probe has been successfully used for the detection and treatment *in vivo*, its focus is on the therapeutic effect of synergistic therapy on tumors. Currently, Near-Infrared probes have been developed for RNA detection. Moreover, the development of fluorescence imaging technology in the Near-Infrared-II window has offered a possibility of *in vivo* imaging with deep penetration and high-fidelity. Luo et al. have presented an NIR-activated nanoprobe for spatiotemporally tunable *in vivo* imaging of miRNA, demonstrating its efficacy for imaging miRNA in living cells. The NIR-activated nanoprobe combines the characteristics of a UV-responsive beacon probe with UCNPs that serve as NIR-to-UV transducers to mediate the wavelength transformation between UV and physiologically active NIR light (Zhao et al., 2020). Moreover, Devaraj et al. constructed novel fluorogenic oligonucleotide probes whose fluorescence is triggered by a vinyl ether tetrazine uncaging process templated by DNA and RNA sequences, proving the application of these probes to detect RNA both *in vitro* and *in vivo*. Applied ICT quenching method has allowed the deployment of multiple fluorophore scaffolds, including NIR-emitting cyanine dyes that are not quenched by tetrazines *via* TBET or FRET mechanisms (Wu et al., 2016). Aside from considering whether the probe can reach the designated site for the detection *in vivo*, the toxicity and metabolism of the probe should also be paid attention to. After the nanoprobe is injected intravenously, it will circulate throughout the body with the blood and accumulate in viscera and brains, so the toxicity of nanoprobes against viscera or brain tissue should be considered more. It is not supposed to ignore the metabolic problems of those with low toxicity, since the accumulation would inevitably occur, resulting in amplification of the toxicity. The potential strategy to solve this problem is to design a probe that can metabolize or to make the probes well targeted, which can reduce the damage of the probe to the human body to a great extent.

## Conclusion

Early diagnosis is the successful detection of tumors before the tumor spreads and becomes incurable, which has attracted a large number of researchers for many years. This review mainly introduces some methods for the detection of tumor-related RNA in recent years, listing several representative examples of DNA-based fluorescent probes and illustrating their advantages and challenges in the detection of tumor-related RNA. The current cancer diagnosis technology, particularly the detection technology of tumor-related RNA, still requires further research and innovation. In the past decade, the research and development of new probes have been the focus of attention, and the detection technology of tumor-related RNA in living cells is relatively mature. Each probe has its unique advantages and disadvantages. In the next research, it is hoped that researchers will pay more attention to the application of probes in practical clinical application rather than just *in situ* detection in cells.
